# Knowledge-Based Planning for Robustly Optimized Intensity-Modulated Proton Therapy of Head and Neck Cancer Patients

**DOI:** 10.3389/fonc.2021.737901

**Published:** 2021-10-19

**Authors:** Yihang Xu, Jonathan Cyriac, Mariluz De Ornelas, Elizabeth Bossart, Kyle Padgett, Michael Butkus, Tejan Diwanji, Stuart Samuels, Michael A. Samuels, Nesrin Dogan

**Affiliations:** Department of Radiation Oncology, University of Miami Miller School of Medicine, Miami, FL, United States

**Keywords:** knowledge-based planning, intensity-modulated proton therapy (IMPT), robust optimization, advanced head and neck cancer, plan quality validation

## Abstract

**Purpose:**

To assess the performance of a proton-specific knowledge-based planning (KBP) model in the creation of robustly optimized intensity-modulated proton therapy (IMPT) plans for treatment of advanced head and neck (HN) cancer patients.

**Methods:**

Seventy-three patients diagnosed with advanced HN cancer previously treated with volumetric modulated arc therapy (VMAT) were selected and replanned with robustly optimized IMPT. A proton-specific KBP model, RapidPlanPT (RPP), was generated using 53 patients (20 unilateral cases and 33 bilateral cases). The remaining 20 patients (10 unilateral and 10 bilateral cases) were used for model validation. The model was validated by comparing the target coverage and organ at risk (OAR) sparing in the RPP-generated IMPT plans with those in the expert plans. To account for the robustness of the plan, all uncertainty scenarios were included in the analysis.

**Results:**

All the RPP plans generated were clinically acceptable. For unilateral cases, RPP plans had higher CTV_primary V100 (1.59% ± 1.24%) but higher homogeneity index (HI) (0.7 ± 0.73) than had the expert plans. In addition, the RPP plans had better ipsilateral cochlea Dmean (−5.76 ± 6.11 Gy), with marginal to no significant difference between RPP plans and expert plans for all other OAR dosimetric indices. For the bilateral cases, the V100 for all clinical target volumes (CTVs) was higher for the RPP plans than for the expert plans, especially the CTV_primary V100 (5.08% ± 3.02%), with no significant difference in the HI. With respect to OAR sparing, RPP plans had a lower spinal cord Dmax (−5.74 ± 5.72 Gy), lower cochlea Dmean (left, −6.05 ± 4.33 Gy; right, −4.84 ± 4.66 Gy), lower left and right parotid V20Gy (left, −6.45% ± 5.32%; right, −6.92% ± 3.45%), and a lower integral dose (−0.19 ± 0.19 Gy). However, RPP plans increased the Dmax in the body outside of CTV (body-CTV) (1.2 ± 1.43 Gy), indicating a slightly higher hotspot produced by the RPP plans.

**Conclusion:**

IMPT plans generated by a broad-scope RPP model have a quality that is, at minimum, comparable with, and at times superior to, that of the expert plans. The RPP plans demonstrated a greater robustness for CTV coverage and better sparing for several OARs.

## Introduction

Head and neck (HN) cancer therapy is both challenging and complicated due to the proximity of clinical target volumes (CTVs) to various critical organs such as the oral cavity, pharynx, larynx, parotids, spinal cord, and brainstem. Radiation therapy for HN cancer is an often used treatment paradigm as an adjuvant to surgery or chemotherapy. Intensity-modulated radiation therapy (IMRT), volumetric modulated arc therapy (VMAT), and intensity-modulated proton therapy (IMPT), all of which can deliver a highly conformal dose to the tumor while sparing organs at risk (OARs), are advanced radiation therapy techniques commonly used for treatment of HN cancer. Both VMAT and IMRT utilize photons to irradiate the patients, while the IMPT utilizes protons. The physical property of proton beams that can eliminate “exit dose” beyond the Bragg peak allows for steeper dose gradients and better OAR sparing than the photon-based therapy. It is well documented that IMPT offers a superior dose distribution as well as reduced toxicity as compared with IMRT and VMAT in the treatment of HN cancers (1, 2). Like IMRT, IMPT utilizes inverse planning optimization to achieve dosimetric objectives. However, the complexity of IMPT planning makes the quality of the IMPT plans very dependent on planner experience and skill, especially for plans in complex anatomy such as the HN region. This may lead to larger variations in plan quality and suboptimal dose distributions (3–5).

Knowledge-based planning (KBP) tools, which incorporate prior treatment planning experience, have the potential to improve the quality and consistency of treatment plans (6–10). One of the commercially available KBP systems [RapidPlan™ (RP) Varian Medical Systems, Palo Alto, CA] employs a dose–volume histogram (DVH) estimation model trained from a library of high-quality treatment plans. It was demonstrated by numerous studies that RP is able to generate IMRT and VMAT plans comparable with or better than the expert plans for a range of treatment sites (11–16). Recently, a proton-specific KBP system [RapidPlanPT™ (RPP), Varian Medical Systems, Palo Alto, CA] was developed to accommodate the physical traits of protons (e.g., no dose beyond the Bragg peak) into the DVH estimation model (17). A small number of publications have explored the usefulness of the RPP for HN cancer. Delaney et al. originally described the principle of RPP and demonstrated the feasibility of generating clinically acceptable planning target volume (PTV)-based IMPT plans by RPP for HN patients (17, 18). In their studies, a relatively narrow scope model was trained and evaluated, where IMPT plans with the same dose prescription and standardize field setup were applied. We believe that more studies are necessary to validate the RPP model reliability before it can be put into clinic use at this early stage. In the present work, we built an RPP model with a wide variety of HN proton plans (e.g., customized field setup, different prescriptions, and both unilateral and bilateral cases). This is a more “broad-scope” model than previously done, and we assessed its performance in the creation of robustly optimized IMPT plans for the HN cancer patients with different dose prescriptions and tumor localization.

## Materials and Methods

### Patient Cohort and Intensity-Modulated Proton Therapy Planning

Seventy-three patients with advanced HN cancer located in the mid/lower HN region, including base of the tongue, tonsil, oropharynx, hypopharynx, parotid, and larynx, were included in this study. These patients were previously treated with VMAT using simultaneous integrated boost (SIB) technique and were enrolled in a retrospective institutional review board (IRB) approved protocol. Thirty of the patients underwent unilateral HN treatment, and the remaining were treated with bilateral HN irradiation. For all patients, contrast and non-contrast planning CTs were acquired in a supine position with 1.5-mm slice thickness using the Siemens Somatom 16 slice CT simulator. All gross tumor volumes (GTVs), CTVs, and OARs, including the spinal cord, brainstem, parotids, constrictors, mandible, cochlea, larynx, carotids, and oral cavity, were delineated on the contrast CT, and these volumes were subsequently transferred to the non-contrast CT. For bilateral treatment, patients were treated with three dose levels: the primary CTV prescribed to 70 Gy; the secondary CTV prescribed to 66, 63, or 60 Gy; and the tertiary CTV prescribed to 56 Gy. For unilateral cases, either one or two dose levels were prescribed with some combination of doses at the levels of 66, 60, 55, 54, and 50 Gy.

For each patient, IMPT plans were generated using multifield optimization (MFO) technique. The IMPT plans employed two to four fields depending on the target extent and anatomy. The field number and arrangement were selected by the expert planners based mainly on the tumor anatomy and location. For each field, a field-specific target was created encompassing all CTVs. These field-specific targets were then modified to avoid having beams entering through the chin area or going through teeth. Streaking artifacts caused by dental implants were delineated and overridden to an appropriate density value. The non-linear universal proton optimizer (NUPO 15.6, Eclipse, Varian Medical Systems) was utilized for optimization along with the proton convolution superposition algorithm (PCS 15.6, Eclipse, Varian Medical Systems) for dose calculation. A relative biological effectiveness (RBE) of 1.1 was used to weight the dose. The spot spacing was set to 0.425 times the energy-dependent in-air full width at half maximum (FWHM) spot size at the isocenter. All IMPT plans were robustly optimized using ±3 mm setup uncertainty (in cardinal directions) along with ±3% proton range uncertainty, resulting in 12 uncertainty scenarios. The targets were the only structures selected to be robustly optimized. The worst-case scenario was required to achieve V95 > 95% (95% of the volume receiving more than 95% of the prescription dose) for the CTVs while keeping the normal tissue constraints as low as possible. The dose constraints used for the OARs are shown in **Table 1**. All plans were normalized such that 95% of the primary CTV volume was covered by the 100% of the prescription dose (V100 = 95%). All proton plans were created by an experienced proton dosimetrist and reviewed by a medical physicist.

**Table 1:** CTV and OAR dose constraints for the nominal IMPT plan.

	Bilateral	Unilateral
CTV	V100 > 95%	
	Dmax < 115%	Dmax < 113%
Brainstem	Dmax < 54 Gy	
Left cochlea	Dmean < 40 Gy	
Right cochlea	Dmean < 40 Gy/50 Gy	
Constrictors	Dmean < 50 Gy	Dmean < 40 Gy
Larynx	Dmean < 50 Gy	Dmean < 30 Gy
Mandible	Dmax < 75 Gy	Dmax < 60 Gy
Oral cavity	Dmean < 50 Gy	Dmean < 30 Gy
Spinal cord	Dmax < 48 Gy	
Left parotid	Dmean < 26 Gy	
	V20 Gy < 50%	
Right parotid	Dmean < 26 Gy	
	V20 Gy < 50%	

V95 represents the relative volume receiving equal or more than the 95% of prescription dose; Dmax represents the maximum dose or relative dose delivered to the structure; V20Gy and Dmean represent the relative volume of the structure receiving more than 20 Gy and mean dose to the volume, respectively.

CTV, clinical treatment volume; OAR, organ at risk; IMPT, intensity-modulated proton therapy.

### Knowledge-Based Planning Model Configuration

The proton-specific KBP optimization tool RPP (Eclipse TPS, ver. 16.1, Varian Medical Systems) was used to create the KBP library. RPP consists of two phases for model configuration: the data extraction phase and the model training phase. In the data extraction phase, the geometric and dosimetric features of selected structures are parameterized for use in model training. During the model training phase, the DVH estimation algorithm is applied to create a DVH estimation model. Individual structure objectives and priorities may be set or generated based on the training set and their principal components. As described in Delaney et al., RPP incorporates a simplified spread-out Bragg peak into the model and utilizes the geometry-based expected dose (GED) metric to estimate the distance of the different voxels in each structure from the target surfaces. Delaney et al. have described RPP modelling in greater detail as well as the differences between the photon-based model and the proton-based model in their work (17), so these details will not be included here.

In our study, 53 IMPT plans consisting of 20 unilateral cases and 33 bilateral HN cases were included in the proton RPP model library. A defined objective list was implemented in the model after initial model training as shown in **Table 2**. The model quality was assessed using model generated plots such as DVH plots, regression and residual plots based on principal component analysis (PCA), and some additional metrics (19). Coefficient of determination (R^2^) and average chi-square (χ^2^) were applied to measure the goodness of fit of the model for each trained OAR, where the R^2^ indicates the correlation between dosimetric and geometric features, while χ^2^ represents the difference between the original and estimated data (19).

**Table 2:** Objectives implemented in the model.

Structure		Relative volume	Absolute/relative dose of specific target prescription	Priority
CTV_primary	Upper	0.0%	102.0%	Generated
	Upper	25.0%	101.0%	Generated
	Lower	100.0%	100.0%	Generated
	Lower	97.0%	100.5%	Generated
	Lower (RO)	95.0%	95.0%	Generated
CTV_secondary	Upper	5%	103%	Generated
	Lower	100%	100%	Generated
	Lower	97%	101%	Generated
	Lower (RO)	95.0%	95.0%	Generated
CTV_tertiary	Upper	5%	103%	Generated
	Lower	100%	100%	Generated
	Lower	97%	101%	Generated
	Lower (RO)	95.0%	95.0%	Generated
Brainstem	Upper	0%	35 Gy	Generated
	Upper	Generated	30% of CTV_primary prescription	Generated
	Upper	Generated	15% of CTV_primary prescription	Generated
Left cochlear	Upper	Generated	25 Gy	Generated
	Mean		5 Gy	Generated
Right cochlear	Upper	Generated	25 Gy	Generated
	Mean		5 Gy	Generated
Constrictor	Upper	Generated	25% of CTV_primary prescription	Generated
	Upper	50%	Generated	Generated
Larynx	Upper	0%	68.5 Gy	Generated
	Mean		53.5 Gy	Generated
Mandible	Upper	0%	101% of CTV_primary prescription	Generated
Oral cavity	Upper	5%	Generated	Generated
	Mean		50 Gy	Generated
Left parotid	Upper	Generated	25% of CTV_primary prescription	Generated
	Upper	50%	Generated	Generated
	Mean		25% of CTV_primary prescription	Generated
Right parotid	Upper	Generated	25% of CTV_primary prescription	Generated
	Upper	50%	Generated	Generated
	Mean		25% of CTV_primary prescription	Generated
Spinal cord	Upper	0%	30 Gy	Generated
	Upper	Generated	30% of CTV_primary prescription	Generated
	Upper	Generated	15% of CTV_primary prescription	Generated
	Upper gEUD		10 Gy	Generated
Spinal cord+3 mm	Upper	0%	35 Gy	Generated
	Upper gEUD		12 Gy	Generated
Submandibular	Mean		26 Gy	Generated

RO, for robust optimization; gEUD, generalized equivalent uniform dose.

### Model Validation

The 20 (10 unilateral cases and 10 bilateral cases) patients who were not included in the model training served as the model-validation group. For each patient used in model validation, RPP plans were created using the same beam arrangement as the corresponding expert plans. Optimization was first performed using an autogenerated objective list by the RPP. One to two additional optimization iterations were performed to improve the CTV coverage or OAR sparing with small changes to the original objective list for some patients if aforementioned dose constraints were not met. The RPP plans were normalized applying the same normalization as the expert plan (V100 = 95%).

The RPP plans were assessed and compared with the expert plans using the same clinical dose–volume constraints for CTVs and OARs. Additionally, we assessed the integral dose deposited in the structure, which removed the CTV volume from the external volume contour (body-CTV). The homogeneity index (HI) was also evaluated for RPP-based IMPT plans and compared with that of the expert plans. In this work, the HI was defined as (20, 21)


$$HI=\frac{D_{2\%}-D_{98\%}}{D_{p}}\times 100$$

where D_2%_ is the dose to 2% of the CTV, D_98%_ is the dose to 98% of the CTV, and D_p_ is the prescription dose for the CTV. The closer the HI value is to zero, the more homogenous the plan is. In order to take the plan robustness into consideration, averaged dosimetric indices over all scenarios (12 uncertainty scenarios plus the nominal scenario) were calculated for each patient, and comparisons was carried out between expert and RPP plans. All comparisons were performed by two-sided Wilcoxon signed-rank test. A p-value <0.05 was considered statistically significant.

## Results

### Model Training Results

**Figure 1:**
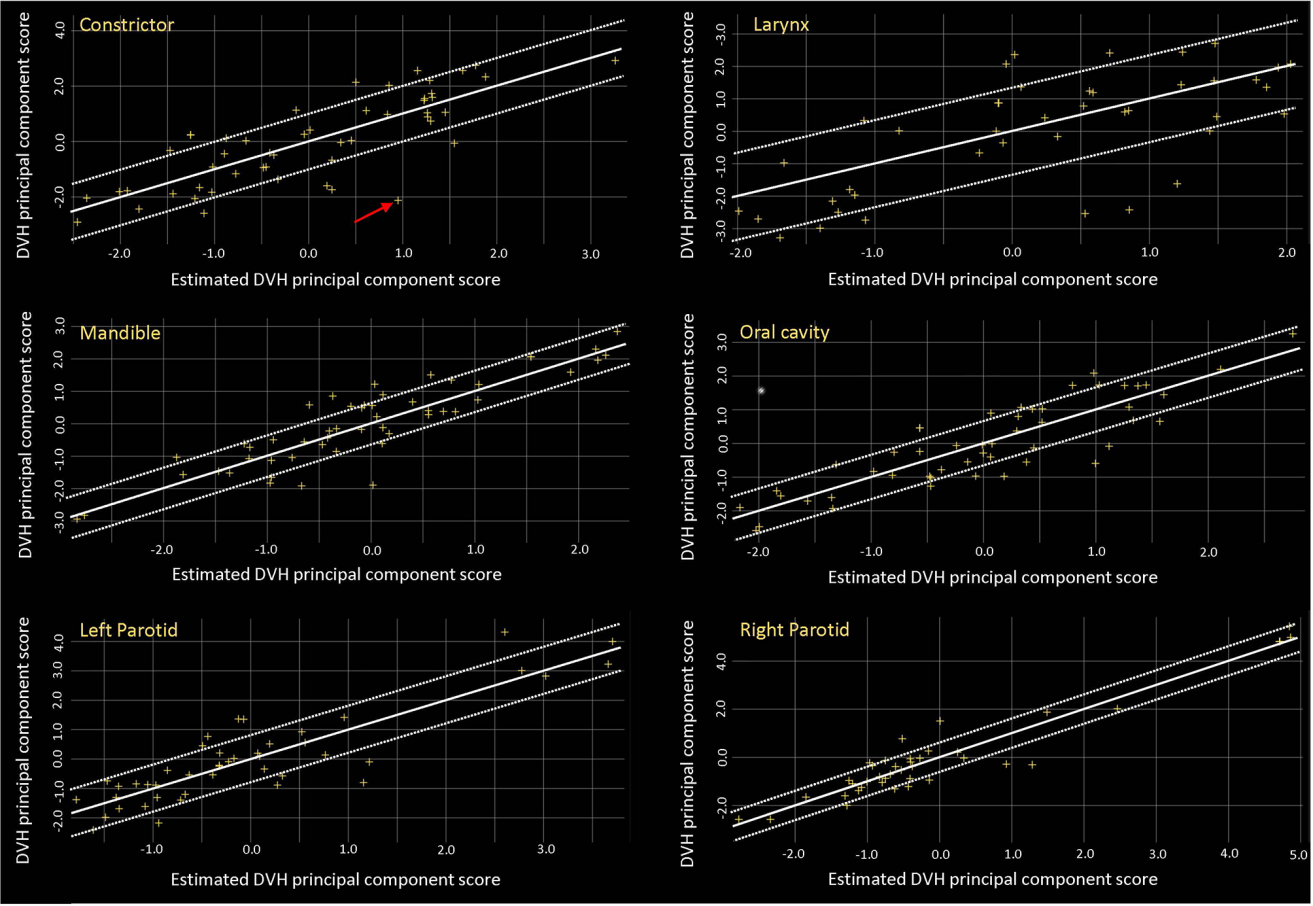
Residual plots for some of the OARs. OARs, organs at risk.

**Table 3** reports the training results for the model. The R^2^ was low for some structures such as the brainstem, larynx, and spinal cord, but the proximity of χ^2^ values (mean ± SD, 1.08 ± 0.02) to 1 indicates that the model is of good quality. **Figure 1** shows the residual plots for some structures. The residual plots show how the original DVH of a structure differs from the estimated DVH, and they were used as a more realistic evaluation of potential influential points that can significantly affect the outcome of the DVH estimation model. Though previous studies have shown that removal of outliers from a good-quality KBP model library with sufficient population often does not have a significant impact on plan quality, outlier cases such as the one marked by the arrow in the constrictor plot were evaluated to determine if the patient needed to be re-planned (12, 22). After review, we believe that they did not need to be excluded, as most outliers were due to an anatomical difference or a difference in the relative location of the object to the CTV; e.g., a large part of constrictor overlapped with the CTV for the arrowed case. Thus, we decided not to remove any of the outliers from the model.

**Table 3:** Model training results.

	Trained number	R^2^	χ^2^
Brainstem	51	0.504	1.062
Left cochlea	50	0.743	1.056
Right cochlea	51	0.727	1.066
Constrictor	51	0.628	1.088
Larynx	44	0.440	1.076
Mandible	51	0.783	1.102
Oral cavity	51	0.742	1.070
Left parotid	53	0.747	1.120
Right parotid	51	0.892	1.103
Spinal cord	53	0.529	1.060
Spinal cord+3 mm	44	0.556	1.066
Submandibular	25	0.973	1.095
CTV_primary	53		
CTV_secondary	47		
CTV_tertiary	36		

### Model Validation Results

**Figure 2:**
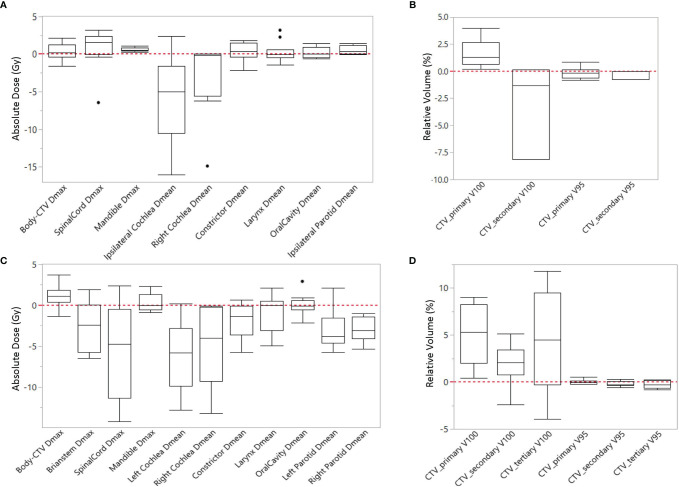
Difference of averaged dose-volume indices over all scenarios between RPP plans and expert plans for **(A, B)** unilateral cases, and **(C, D)** bilateral cases.

Most IMPT plans generated by the expert planners and RPP met the clinical constraints in **Table 1**. Some constraints were not met for very few cases due to close proximity of some OARs to CTVs. After review on these cases, these plans were clinically acceptable. **Table 4** summarizes the comparison of dosimetric indices presented as mean ± SD between the RPP-generated plans and the expert plans for 10 unilateral (**Table 4A**) and 10 bilateral (**Table 4B**) cases in the validation group. The range of each dosimetric index was also presented in brackets as (min, max) in **Table 4**. The dosimetric indices from nominal plans as well as the averaged dosimetric indices over all scenarios are listed in **Table 4**. To take the plan robustness into consideration, we will only focus on the results of averaged dosimetric indices over all scenarios. **Figures 2A, B** show the difference of averaged dose–volume indices over all scenarios between the RPP and expert plans for unilateral cases, and **Figures 2C, D** show the differences for bilateral cases.

**Table 4A:** Dosimetric comparison between RPP plans and expert plans for 10 unilateral cases in the validation group.

	Nominal plan				All scenarios averaged			
	Expert	RPP	RPP − Expert	p-Value	Expert	RPP	RPP − Expert	p-Value
CTV_primary V100 (%)	95 ± 0	95 ± 0	0 ± 0	1.000	89.69 ± 2.62 (86.18, 93.23)	91.28 ± 2.27 (86.4, 93.45)	1.59 ± 1.24	0.002
CTV_primary V95 (%)	99.74 ± 0.27 (99.13, 100)	99.43 ± 0.41 (98.65, 100)	−0.3 ± 0.29	0.012	98.99 ± 0.84 (97.03, 100)	98.83 ± 0.6 (97.87, 100)	−0.15 ± 0.53	0.426
CTV_primary HI	5.81 ± 1.31 (2.57, 7.39)	6.53 ± 1.56 (2.98, 9.04)	0.73 ± 0.48	0.002	8.01 ± 2.68 (3.16, 13.48)	8.71 ± 2.72 (3.51, 14.07)	0.7 ± 0.73	0.020
CTV_secondary V100 (%)	99.55 ± 0.73 (98.71, 99.97)	97.44 ± 2.63 (94.7, 99.95)	−2.11 ± 2.78	0.250	98.05 ± 1.85 (95.99, 99.57)	94.97 ± 3.68 (91.43, 98.78)	−3.09 ± 4.43	0.500
CTV_secondary V95 (%)	99.96 ± 0.08 (99.87, 100)	99.79 ± 0.18 (99.65, 100)	−0.16 ± 0.18	0.500	99.64 ± 0.44 (99.13, 99.93)	99.39 ± 0.48 (99.09, 99.94))	−0.25 ± 0.44	1.000
Brainstem Dmax (Gy)	9.02 ± 8.42 (1.15, 26.01)	8.01 ± 6.31 (0.97, 18.96)	−1.01 ± 3.22	0.734	9.29 ± 8.37 (1.08, 26.02)	8.41 ± 6.39 (0.92, 19.4)	−0.88 ± 3.14	0.652
Spinal cord Dmax (Gy)	14 ± 8.15 (1.35, 25.11)	14.73 ± 7.5 (0.97, 26.65)	0.73 ± 2.92	0.106	14.51 ± 8.05 (1.78, 25.34)	15.17 ± 7.34 (3.99, 26.8)	0.66 ± 2.77	0.160
Mandible Dmax (Gy)	60 ± 5.95 (50.69, 69.35)	60.8 ± 6.02 (50.92, 70.01)	0.81 ± 0.6	0.008	60.09 ± 5.78 (50.75, 68.72)	60.71 ± 5.8 (51.06, 69.26)	0.62 ± 0.25	0.004
Ipsilateral cochlea Dmean (Gy)	15.45 ± 12.25 (1.06, 39.28)	9.57 ± 6.82 (0.9, 23.01)	−5.89 ± 6.19	0.012	15.62 ± 12.23 (1.16, 39.61)	9.86 ± 6.86 (1, 23.56)	−5.76 ± 6.11	0.012
Constrictor Dmean (Gy)	12 ± 8.93 (3.74, 32.31)	12.41 ± 9.22 (3.49, 33.97)	0.41 ± 1.29	0.426	12.06 ± 8.86 (3.92, 32.25)	12.47 ± 9.13 (3.66, 33.83)	0.41 ± 1.28	0.426
Larynx Dmean (Gy)	5.21 ± 6.48 (0.01, 15.85)	5.5 ± 7.31 (0.01, 19.15)	0.29 ± 1.42	0.770	5.3 ± 6.57 (0.01, 16.11)	5.58 ± 7.37 (0.01, 19.26)	0.27 ± 1.38	0.770
Oral cavity Dmean (Gy)	4.17 ± 5.31 (0, 17.4)	4.46 ± 5.13 (0, 16.8)	0.29 ± 0.73	0.375	4.23 ± 5.31 (0, 17.41)	4.51 ± 5.13 (0, 16.82)	0.28 ± 0.73	0.375
Ipsilateral parotid Dmean (Gy)	20.1 ± 14.73 (3.32, 38.86)	20.61 ± 14.16 (4.74, 38.84)	0.51 ± 0.63	0.250	20.15 ± 14.72 (3.4, 38.87)	20.67 ± 14.14 (4.81, 38.85)	0.52 ± 0.63	0.250
Ipsilateral parotid V20Gy (%)	42.62 ± 32.94 (6.02, 85.26)	43.77 ± 31.93 (8.98, 85.59)	1.15 ± 1.22	0.125	42.67 ± 32.84 (6.12, 85.07)	43.82 ± 31.75 (9.09, 85.26)	1.14 ± 1.26	0.125
Body-CTV Dmax (Gy)	63.55 ± 5.25 (53.06, 70.57)	64.26 ± 4.62 (55.75, 70.82)	0.71 ± 1.66	0.322	64.19 ± 5.12 (54.27, 71.83)	64.53 ± 4.74 (56.44, 71.97)	0.33 ± 1.07	0.375
Body-CTV Dmean (Gy)	1.72 ± 0.93 (0.74, 3.37)	1.71 ± 0.93 (0.82, 3.36)	−0.01 ± 0.08	0.846	1.72 ± 0.93 (0.74, 3.38)	1.71 ± 0.93 (0.82, 3.37)	−0.01 ± 0.08	0.846

The data are presented as mean ± standard deviation, and the range is presented in brackets as (min, max). Only two of 10 unilateral patients contained CTV_secondary.

RPP, RapidPlanPT.

The red-colored values indicate statistically significant difference (p-Value< 0.05).

**Table 4B:** Dosimetric comparison between RPP plans and expert plans for 10 bilateral cases in the validation group.

	Nominal plan				All scenarios averaged			
	Expert	RPP	RPP − Expert	p-Value	Expert	RPP	RPP − Expert	p-Value
CTV_primary V100 (%)	95 ± 0	95 ± 0	0 ± 0	1.000	82.03 ± 2.72(77.87, 87.7)	87.1 ± 3.06(81.65, 89.92)	5.08 ± 3.02	0.002
CTV_primary V95 (%)	99.86 ± 0.42(98.66, 100)	99.83 ± 0.42(98.63, 100)	−0.04 ± 0.04	0.008	99.52 ± 0.67(97.7, 99.91)	99.51 ± 0.51(98.23, 99.94)	−0.01 ± 0.24	0.625
CTV_primary HI	4.04 ± 1.44(3.22, 8.01)	4.39 ± 1.64(2.68, 8.71)	0.35 ± 0.47	0.027	6.63 ± 2.23(5.16, 12.77)	6.22 ± 2.11(4.55, 11.46)	−0.4 ± 0.77	0.131
CTV_secondary V100 (%)	95.15 ± 1.76(92.44, 97.44)	95.85 ± 2.28(91.03, 98.68)	0.7 ± 2.01	0.160	91.26 ± 2.91(86.67, 94.61)	93.18 ± 3.53(84.23, 96.15)	1.92 ± 2.19	0.037
CTV_secondary V95 (%)	99.82 ± 0.45(98.54, 99.99)	99.65 ± 0.44(98.49, 99.93)	−0.17 ± 0.17	0.004	99.37 ± 0.59(97.82, 99.83)	99.21 ± 0.52(97.95, 99.74)	−0.16 ± 0.27	0.160
CTV_tertiary V100 (%)	95.97 ± 1.97(92.02, 98.99)	96.8 ± 2.08(92.51, 99.45)	0.83 ± 2.69	0.846	88.83 ± 3.28(83, 94.33)	92.92 ± 3.62(85.09, 98.01)	4.09 ± 5.33	0.037
CTV_tertiary V95 (%)	99.87 ± 0.13(99.65, 99.99)	99.62 ± 0.25(99.21, 99.92)	−0.26 ± 0.21	0.002	99.34 ± 0.31(98.77, 99.75)	99.08 ± 0.43(98.55, 99.7)	−0.26 ± 0.4	0.131
Brainstem Dmax (Gy)	35.25 ± 3.89(28, 41.49)	33.08 ± 4.03(28.64, 40.75)	−2.17 ± 3.05	0.084	35.81 ± 3.79(28.65, 41.59)	33.32 ± 3.99(29.3, 41.24)	−2.48 ± 3.04	0.035
Spinal cord Dmax (Gy)	41.86 ± 3.01(35.59, 45.68)	36.13 ± 4.52(27.1, 41.49)	−5.73 ± 5.62	0.006	42.37 ± 3.13(36.07, 45.81)	36.9 ± 4.43(28.78, 43)	−5.47 ± 5.72	0.014
Mandible Dmax (Gy)	70.63 ± 3.33(61.87, 74.2)	70.8 ± 3.93(61.32, 75.02)	0.18 ± 1.16	0.865	70.43 ± 3.36(61.78, 74.24)	70.77 ± 4.01(61.16, 75.02)	0.34 ± 1.06	0.625
Left cochlea Dmean (Gy)	13.61 ± 5.17(1.87, 19.63)	7.38 ± 2.94(2.06, 13.28)	−6.23 ± 4.43	0.004	13.67 ± 5.12(2.04, 19.59)	7.62 ± 2.98(2.25, 13.67)	−6.05 ± 4.33	0.006
Right cochlea Dmean (Gy)	13.15 ± 5.84(2.95, 18.4)	8.24 ± 4.13(2.73, 15.62)	−4.91 ± 4.79	0.002	13.25 ± 5.77(3.03, 18.4)	8.41 ± 4.03(2.83, 15.54)	−4.84 ± 4.66	0.002
Constrictor Dmean (Gy)	48.72 ± 1.76(45.43, 51.34)	46.75 ± 3.57(41.33, 50.88)	−1.97 ± 2.32	0.049	48.84 ± 1.77(45.43, 51.52)	46.93 ± 3.52(41.63, 51.08)	−1.91 ± 2.27	0.049
Larynx Dmean (Gy)	44.44 ± 2.76(39.94, 48.67)	43.14 ± 3.46(37.66, 47.85)	−1.01 ± 2.34	0.297	44.69 ± 2.79(40.12, 48.96)	43.46 ± 3.47(37.89, 48.18)	−0.96 ± 2.29	0.375
Oral cavity Dmean (Gy)	20.61 ± 15.36(5.19, 49.14)	20.69 ± 15.54(4.68, 49.14)	0.07 ± 1.17	0.846	20.62 ± 15.29(5.24, 48.99)	20.69 ± 15.47(4.74, 48.98)	0.07 ± 1.16	0.922
Left parotid Dmean (Gy)	22.37 ± 1.07(20.05, 23.71)	19.35 ± 3.14(14.08, 25.03)	−3.01 ± 2.39	0.014	22.66 ± 1.16(19.99, 24.24)	19.72 ± 3.19(14.26, 25.31)	−2.94 ± 2.34	0.014
Left parotid V20Gy (%)	44.74 ± 2.04(41.86, 47.92)	38.11 ± 6.14(27.83, 46.76)	−6.63 ± 5.43	0.006	45.16 ± 2.21(42.32, 48.84)	38.71 ± 6.19(28.11, 47.13)	−6.45 ± 5.32	0.010
Right parotid Dmean (Gy)	20.88 ± 2.45(15.23, 23.09)	17.88 ± 3.16(10.84, 21.82)	−3 ± 1.46	0.002	21.17 ± 2.56(15.22, 23.65)	18.23 ± 3.24(10.95, 22.4)	−2.94 ± 1.42	0.002
Right parotid V20Gy (%)	42.62 ± 5.14(29.26, 49.01)	35.63 ± 6.71(19.24, 42.55)	−6.98 ± 3.45	0.002	43.18 ± 5.32(29.29, 49.71)	36.26 ± 6.83(19.48, 43.48)	−6.92 ± 3.45	0.002
Body-CTV Dmax (Gy)	73 ± 1.13(71.52, 75.2)	74.69 ± 1.21(73.02, 77.19)	1.69 ± 1.39	0.008	73.85 ± 1.28(72.39, 76.82)	75.04 ± 1.21(73.43, 77.23)	1.2 ± 1.43	0.049
Body-CTV Dmean (Gy)	5.59 ± 0.95(4.68, 7.25)	5.4 ± 1.04(4.45, 7.12)	−0.19 ± 0.19	0.014	5.59 ± 0.96(4.68, 7.25)	5.4 ± 1.04(4.45, 7.13)	−0.19 ± 0.19	0.014

The data are presented as mean ± standard deviation, and the range is presented in brackets as (min max).

RPP, RapidPlanPT.

The red-colored values indicate statistically significant difference (p-Value< 0.05).

In unilateral cases, RPP plans achieved more robust CTV coverage with a moderately higher CTV_primary V100 (1.59% ± 1.24%), whereas the expert plans were more homogeneous with a slightly lower CTV_primary HI (0.7 ± 0.73) than the RPP plans. The Dmax of the mandible from RPP plans was marginally higher than that of the expert plans (0.62 ± 0.25 Gy), but the RPP plans had a better ipsilateral cochlea Dmean (−5.76 ± 6.11 Gy). For other OAR dose–volume indices, there was no statistically significant difference between RPP and expert plans. In the bilateral cases, the V100 for all CTVs prescribed with different dose levels was higher for the RPP plans, especially for CTV_primary V100 (5.08% ± 3.02%), indicating that RPP plans were more robust for CTV coverage. There was no statistically significant difference for the CTV_primary HI between the expert and RPP plans. With respect to OAR sparing, RPP plans had significantly superior dosimetric indices for several OARs such as the spinal cord Dmax (−5.74 ± 5.72 Gy), the left and right cochlear Dmean (left cochlea, −6.05 ± 4.33 Gy; right cochlea, −4.84 ± 4.66 Gy), the left and right parotid V20Gy (left parotid, −6.45% ± 5.32%; right parotid, −6.92% ± 3.45%), and the integral dose (−0.19 ± 0.19 Gy). However, RPP plans increased the Dmax for the body (body-CTV) (1.2 ± 1.43), revealing that the RPP plans produced a slightly higher hotspot in normal tissue than the expert plans. In this study, only patients with HN cancers in low/mid region (e.g., laryngeal to tonsillar lesions) were included. Therefore, eyes, optic nerves, and optic chiasm received relatively low doses when compared with our clinical dose constraints (Dmax < 45 Gy). For eyes, the Dmax was less than 3.5 Gy for all cases; and for optic nerves and chiasm, the Dmax was always less than 0.5 Gy except for one unilateral case where the left optic nerve had a Dmax = 7.15 Gy in the expert plan and 6.34 Gy in the RPP plan.

**Figure 3:**
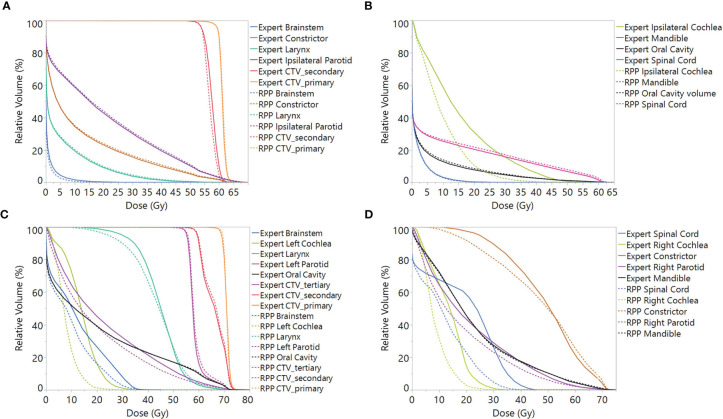
Mean DVH including all scenarios over validation patients for **(A, B)** unilateral cases and **(C, D)** bilateral cases. Note: In order to average the CTV DVH over all patients, for each unilateral case, the DVH of CTV_primary was normalized to V60Gy = 95%, and the DVHs of CTV_secondary was normalized to V54Gy = 95%, while for bilateral cases, the CTV_secondary was normalized to V60Gy = 95%. DVH, dose–volume histogram; CTV, clinical treatment volume.

**Figures 3A** and **3B** present the mean DVH, including all scenarios over 10 unilateral patients from the validation group, and **Figures 3C, D** present the mean DVH over 10 bilateral patients. It can be observed that for unilateral cases, the RPP plans had comparable DVH with the expert plans, though the RPP plans achieved a superior DVH for the ipsilateral cochlea DVH. Concerning the DVH of bilateral cases, the RPP plans did a better job of sparing the brainstem, spinal cord, cochlea, and parotids.

**Figure 4:**
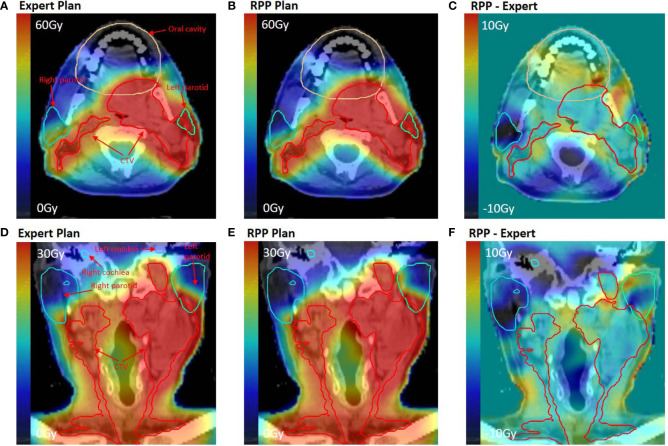
Dose distribution for an example bilateral case in the validation group. **(A, D)** Dose distribution of the expert plan. **(B, E)** Dose distribution of the RPP plan. **(C, F)** Dose difference map between RPP and expert plan. **(A–C)** On axial plane, while **(D–F)** on coronal plane.

**Figure 4** shows the dose distributions of the RPP and expert plans for an example bilateral case from the validation group. **Figures 4A–C** indicate that although the RPP delivered a slightly higher dose to the oral cavity (oral cavity Dmean = 25.99 *vs.* 23.62 Gy), the RPP achieved better sparing for parotids, especially for right parotid (left parotid Dmean = 22.96 *vs.* 25.03 Gy, right parotid Dmean = 10.84 *vs.* 15.23 Gy). In **Figures 4D** and **E**, both the RPP and expert plans met the constraints for cochlea (Dmean < 40 Gy), but the RPP plan achieved much better sparing than the expert plan (left cochlea Dmean = 13.28 *vs.* 17.52 Gy, right cochlea Dmean = 4.7 *vs.* 18.21 Gy).

## Discussion

This work demonstrated that a proton-specific KBP model, RPP, can generate high-quality IMPT plans for the HN cancer patients. One of the benefits of employing RPP is its high efficiency. On average, it required about 20 min to generate the prediction, optimizations, and dose calculation when utilizing the RPP model. In comparison, it typically took more than 2 h to complete HN IMPT plans by experienced dosimetrists in our study. Moreover, our results indicate that the plans generated by the RPP have greater robustness with respect to the CTV coverage when certain uncertainty parameters (3% range uncertainty and 3-mm setup uncertainty) were applied, which is consistent with the results from our previous study (23). For the unilateral cases, the RPP plans achieved comparable OAR sparing with the expert plans except for the ipsilateral cochlea where the RPP plans delivered lower dose. Regarding bilateral cases, the RPP plans improved the sparing in brainstem, spinal cord, cochlea, parotids, and constrictors without reducing the plan homogeneity when compared with the expert plans. In addition, the reduction of integral dose, which is one of the main advantages of proton therapy, was observed in the RPP plans compared with the expert plans for bilateral cases. As a tradeoff, the bilateral RPP plans produced a slightly higher hotspot than the expert plans in the body outside of CTVs (1.69 ± 1.39 Gy). In general, our results are consistent with the previous studies illustrating that the RPP plans were at least equivalent to if not better than the expert plans (18, 24, 25).

Different from the earlier studies by Delaney et al., which employed cases with the same prescription and standardized beam arrangement for model training and validation (17, 18), our model was more broad-scope in that it included cases prescribed with varying dose levels and using different customized beam arrangements. The results suggest that this broad-scope model can create IMPT plans of good quality in the HN regardless of the beam arrangement and prescription. It has been demonstrated that the quality of VMAT plans for HN cancer created by the RP model was independent of prescription and beam geometry (26). A study comparing a proton model trained with customized beam number and arrangement to another model trained with standardized beam number and arrangement for hepatocellular carcinoma treatment indicated that two models performed equivalently with no statistically significant difference for almost all dose–volume parameters (24). However, as the quality of the proton plans is more dependent on the beam arrangement than the photon plans, the impact of employing IMPT plans with different beam arrangements *versus* standardized beam arrangement in the model should be investigated for HN IMPT model. Future work on the integration of an automated beam angle selection algorithm, which is under investigation (27), should be done to see if there can be further improvement in plan quality and efficiency.

Concerning the treatment area, this study combined both unilateral and bilateral cases in the model training. In some photon KBP studies, models trained by combining unilateral and bilateral plans showed high quality in treatment of HN cancer (28). Another investigation revealed that a photon model trained by unilateral cases was able to generate high-quality VMAT plans for bilateral breast treatments (29). It is yet not clear whether a combined model or a specific model is better in generation of HN IMPT plans. In photon therapy, one study showed that specific model resulted in improved quality for liver cancer (30), while another study revealed that there was no difference of the quality between a specific model and a combined model for prostate cancer (31). Therefore, it is worthwhile to explore whether there is any benefit of utilizing specific models by separating unilateral and bilateral cases for IMPT plan generation in HN cancer treatment.

This study included 53 cases for model training, and no outlier was removed from the model. Potential outliers identified by the RPP system indicate that the plan has a statistically significant difference as compared with the whole population in the model. However, earlier studies by Delaney et al. and Hussein et al. compared the quality of the plans generated by an outlier-free model to a model without outlier removal and demonstrated that the impact of a small number of outliers does not significantly impact the plan quality (12, 22). Our previous investigation by Bossart et al. (31) also showed that the differences between refined KBP model generated by eliminating the dosimetric outliers and the original KBP generated plans were insignificant. According to the results, we believe that 53 patients should be enough to generate a reliable model, but it would be necessary to investigate the influence of the model size on the IMPT plan quality.

One limitation of this study is that the 20 patients included in the validation set consisted of 10 unilateral cases and 10 bilateral cases, which may not be sufficient to confirm the reliability of the model, as it is at early stage for RPP exploration. That being said, many publications on KBP photon and proton models have included small numbers of plans for validation, and the vendor’s recommendation is 10 validation cases to prove the model is working sufficiently (12, 16, 17, 24, 31, 32).

## Conclusion

This work explored the performance of a broad-scope proton-specific KBP model to generate robustly optimized IMPT plans for HN cancer patients. The results demonstrated that the IMPT plans created by the model have high quality that is at least comparable and even, in some ways, superior to that of the expert plans. The IMPT plans generated by the model had greater robustness for CTV coverage and better sparing for several OARs. More studies should be done to evaluate the RPP model reliability.

## Data Availability Statement

The original contributions presented in the study are included in the article/supplementary material. Further inquiries can be directed to the corresponding author.

## Ethics Statement

The studies involving human participants were reviewed and approved by University of Miami Institutional Review Board. Written informed consent for participation was not required for this study in accordance with the national legislation and the institutional requirements.

## Author Contributions

ND designed the study, oversaw the whole study, and trained and validated the model library. YX carried out all data analysis and wrote the manuscript. JC, MO, and MB created IMPT expert plans for model training and validation. EB provided expertise on the proton model training and validation. KP provided his expertise in data analysis. TD provided his expertise in the study design and evaluated the IMPT plans. SS and MS provided contouring of the cases included in this study and reviewed the assessment of the cases. All authors contributed to the article and approved the submitted version.

## Funding

This work was supported in part by a research grant from Varian Medical Systems, Palo Alto, CA (GR013242).

## Conflict of Interest

The authors declare that the research was conducted in the absence of any commercial or financial relationships that could be construed as a potential conflict of interest.

## Publisher’s Note

All claims expressed in this article are solely those of the authors and do not necessarily represent those of their affiliated organizations, or those of the publisher, the editors and the reviewers. Any product that may be evaluated in this article, or claim that may be made by its manufacturer, is not guaranteed or endorsed by the publisher.
